# Working Smarter Not Harder: Oxytocin Increases Domestic Dogs’ (*Canis familiaris*) Accuracy, but Not Attempts, on an Object Choice Task

**DOI:** 10.3389/fpsyg.2019.02141

**Published:** 2019-10-01

**Authors:** Jessica Lee Oliva, Manuel Mengoli, Tiago Mendonça, Alessandro Cozzi, Patrick Pageat, Camille Chabaud, Eva Teruel, Céline Lafont-Lecuelle, Cécile Bienboire-Frosini

**Affiliations:** ^1^Research Institute in Semiochemistry and Applied Ethology (IRSEA), Apt, France; ^2^Clinical Ethology and Animal Welfare Centre (CECBA), Apt, France

**Keywords:** oxytocin, DAP, dog, attachment, object choice, pheromone, cognition, OCT

## Abstract

The neuropeptide oxytocin (OT) has been shown to enhance dogs’ ability to perform an object choice task (OCT) involving the use of human pointing cues, when delivered intranasally. This study aimed at further investigating whether OT enhances task performance by increasing choices made, or by increasing correctness of choices made, and to compare these treatment effects to dog appeasing pheromone (DAP), known to balance emotional activation in dogs. Hence, we compared OCT performance between three groups of dogs: (i) dogs administered OT and a sham collar, (ii) dogs administered a saline placebo and a DAP collar, and (iii) control dogs administered a saline placebo and a sham collar. All three groups consisted of a combination of male and female pet dogs and assistance-dogs-in-training currently living with a volunteer carer. The study also evaluated the effect of intranasal OT and/or DAP on plasma levels of OT, and prolactin; which has previously been linked with anxiety in dogs. The dogs’ emotional state was measured using the Emotional Disorders Evaluation in Dogs (EDED) scale. The owners’/carers’ degree of anxious- and avoidant-style attachment to their dogs was accessed using the Pet Attachment Questionnaire (PAQ). Interesting descriptive data appeared for both treatment groups. Particularly, in OT group, we obtained significant results demonstrating that intranasal OT enhances OCT performance in dogs compared to control, by increasing the percentage of correct choices, but not the number of choices, made. Results also support that the mode of action of intranasal OT is via direct access to the brain and not via the blood, since no elevation of plasma OT (or prolactin) levels were observed after intranasal administration in this study. Similarly, DAP application did not significantly alter OT or prolactin peripheral concentrations. Several differences were observed between fostered and pet dogs, namely: fostered dogs demonstrated higher levels of serum prolactin, made more choices on the OCT compared to pet dogs but were not more likely to be correct, and were fostered by carers with higher avoidant attachment scores than pet dog owners. These findings implicate consideration of potential carer and training consequences for assistance dogs.

## Introduction

Dogs have remarkable abilities to communicate with humans (Hare and Tomasello, 2005), and we now specifically breed dogs for the unique purpose of placing them in working roles that assist humans. Nowadays, dogs are used by humans for various working roles, including assisting, guiding, herding, detecting, racing, and guarding (Cobb et al., 2015). The ability of dogs to successfully work in these roles is dependent on a wide variety of factors depending on the job, but for assistance (guiding, hearing, and service) dogs (for definition see, Bremhorst et al., 2018), this includes their ability to cope in stressful situations, and to form affiliative bonds with their human counterparts. This is no small feat as it is often necessary for these dogs to forge several human relationships over their lifetime, each time detaching from the previous to allow the new relationship to form. If they turn out to be mismatched to their handler, they can be re-homed up to eight times (Lloyd et al., 2016) and must detach and attach every time. Cobb et al. (2015) has estimated the current rate of success across a variety of trained working dogs to be only around 50%. The total cost of a guide dog’s 8-year working life has been estimated to be US$40,598 with the majority of these costs (US$34,972) incurred by the training school during the dog’s first year of life (Wirth and Rein, 2008). Hence, research to close this gap between steep costs and low success rates is clearly warranted.

Dog-human attachment should not be considered in one direction only – the ability of humans to attach to a working dog is also very important to consider. Following the work of Ainsworth et al. (1978) who classified human infants as securely, anxiously or avoidantly attached to their primary caregiver, Zilcha-Mano et al. (2011) found that these styles of attachment can also be applied to the way in which adult owners attach to their pets. The fact that assistance dogs are raised by volunteer carers who inevitably must give them back to the assistance dog association in which they were born may cause carers to form more insecure (anxious and/or avoidant) attachments with the dogs they care for, compared to companion dog owners. Avoidant adult attachment styles have been associated with owning dogs with separation-related disorders (Konok et al., 2015), and this could be quite problematic in a foster care situation where there would be an even greater need for the carer to guard against an inevitable loss. While previous work by Mariti et al. (2013) showed that there was no difference in behaviors indicating an attachment bond between pets and search-and-rescue dogs who live with their “handlers,” these differences have not been investigated in pets versus fostered dogs.

Insecure attachments have been shown to impact dogs’ ability to use human social gestures. For example, Oliva et al. (2016a) showed that owners who scored high for anxious attachments to their dogs, according to the Pet Attachment Questionnaire (PAQ) (Zilcha-Mano et al., 2011), owned dogs that were more likely to perform poorer on an object choice task (OCT) in which pointing cues were used to indicate the location of a hidden food reward. For dogs whose job requires the use of human pointing gestures or the ability to read human non-verbal communication in general, this could be a critical problem. Interestingly, this relationship disappeared following the intranasal administration of synthetic oxytocin (OT) (Oliva et al., 2016a). Oxytocin is known for its role in mammalian bonding (for a review see, Lim and Young, 2006) and social cognition in humans (for a review see, Bartz et al., 2011). In dogs, intranasally applied OT has been shown to increase positive expectations (Kis et al., 2015), decrease friendliness in response to a threatening person (Hernádi et al., 2015), increase affiliation toward owners and conspecifics (Romero et al., 2014), increase play behaviors toward conspecifics (Romero et al., 2015) and increase mutual gaze with their owners (Nagasawa et al., 2015). It has also been found to enhance performance on OCTs (Oliva et al., 2015; Macchitella et al., 2017). It remains unknown exactly how and where OT exerts its OCT-enhancing effects. With regards to “how,” two possibilities exist. The first is that OT *directly* improves social cognitive function, and the second is that it *indirectly* enhances cognitive function by modulating emotions which may be inhibiting social cognition. Indeed, in humans, OT has been shown to have anxiolytic properties (Heinrichs et al., 2003; de Oliveira et al., 2012). With regards to “where,” it has long been believed that intranasally administered peptides gain access to the brain as increased levels of the peptide can be measured in cerebrospinal fluid (CSF) following administration (Born et al., 2002; Gossen et al., 2012; Neumann et al., 2013; Striepens et al., 2013; Dal Monte et al., 2014; Freeman et al., 2016; Rault, 2016; Lee et al., 2018). However, as discussed in their review, Leng and Ludwig (2016) explain that the increases in CSF are only modest in comparison to the amount administered. However, the authors also acknowledge that central OT may be largely degraded in brain tissue and therefore only enter the CSF in minimal amounts. Nevertheless, the relatively large rise in peripheral measures that sometimes follows intranasal application has cast doubt in these researchers’ minds that the administered peptide is primarily acting at the level of the brain (Leng and Ludwig, 2016). Furthermore, it has recently been suggested that the neuro-behavioral effects of intranasally administered OT may stem from a peripheral mechanism of action, following results from a study by Lee et al. (2018) which demonstrated highly variable timings and extents that both intranasally and intravenously administered OT reached the CSF of monkeys. Temesi et al. (2017) and Romero et al. (2014) also demonstrated an increase in OT measured in dog blood following intranasal application, however, as these studies used varying methods, doses, and small sample sizes, the current study will extend upon these findings in a larger sample of dogs using recently validated methods (Bienboire-Frosini et al., 2017; MacLean et al., 2017), as stated hereunder in the “Materials and Methods” section.

Unfortunately, central OT function is difficult to measure in a minimally invasive way. Indeed, OT is produced in the hypothalamus and released from magnocellular neurons that project to the posterior lobe of the pituitary where it is naturally secreted into the bloodstream and acts as a hormone (Lim and Young, 2006). Oxytocin is also directly secreted into central brain regions from hypothalamic magnocellular neurons (Knobloch et al., 2012) which also release the peptide into the CSF from their somas and dendrites, where it has the potential to act as a kind of slower-acting and longer-lasting “hormone” in the central nervous system (Ludwig and Leng, 2006). Extrapolating from peripheral measures is problematic because the central and peripheral release of OT from magnocellular neurons of the hypothalamus are not necessarily time-locked; OT can be released independently at different neuronal sites, in response to the same stimulus. Indeed, there are reports of dendritic release being delayed by more than 1 h, compared to the more immediate release at the terminal bouton, and exerting its effects for much longer (Ludwig and Leng, 2006). Furthermore, certain social stressors in rats have been shown to induce central, but not peripheral secretion (Engelmann et al., 1999). Conversely, studies in guinea pigs show that peripheral OT increases in response to suckling, without an accompanying increase in the CSF [measured by immunoassay with prior solid phase extraction (Amico et al., 1990) and High-Performance Liquid Chromatography (Robinson and Jones, 1982)]. As neuropeptides do not readily pass the blood–brain barrier (Vorherr et al., 1968; Mens et al., 1983; Robinson, 1983; Veening et al., 2010), it follows that levels in the brain and in the blood may be substantially different at any one time.

Thus, while extrapolating from peripheral measures to draw conclusions about real-time central OT function is dubious, peripheral levels of the peptide may be a possible reflection of the overall functioning of the oxytocinergic system of the organism. For example, in humans, plasma OT is positively associated with number of attachment figures (Jobst et al., 2014) and tendency to be emotionally open (Tops et al., 2007). For this reason, we are interested in investigating the association between plasma levels of OT and OCT performance in dogs, and the influence of intranasally administered OT, in the current study. We are also interested in measuring serum prolactin, firstly because its release can be stimulated by OT, for example in response to suckling, mating and ovarian steroids (Kennett and McKee, 2012), but also because it is released in response to situational stressors in humans (Jeffcoate et al., 1986; Armario et al., 1996) and rats (Torner and Neumann, 2002; Torner et al., 2004). In addition, serum prolactin has been associated with scores on the Emotional Disorders Evaluation in Dogs (EDED; Pageat, 1995) scale in a sample of anxious dogs (Pageat et al., 2007). The EDED assesses behaviors and physiologies of dogs that are modified by emotional disorders and scores them according to their severity.

An alternative method to potentially activate central OT is via the administration of pheromones believed to involve the release of OT in the brains of mammals (see reviews, Bielsky and Young, 2004; Wacker and Ludwig, 2012). In dogs, a synthetic analog of the natural maternal appeasing pheromone, known as dog appeasing pheromone (DAP) (ADAPTIL^®^; Ceva Santé Animale), which is naturally released by bitches to appease their young, has been identified. Processing of pheromones occurs within the medial amygdala (Salazar and Sànchez Quintero, 2009) and is likely to integrate information coming from both the main olfactory bulb and the accessory olfactory bulb, where OT receptors have been reported (Wacker and Ludwig, 2012). Behavioral effects of DAP are not dissimilar to the behavioral effects following intranasal OT administration. For example, studies have shown that DAP reduces behavioral indicators of stress in a shelter (Tod et al., 2005), owner-reported fear of fireworks (Sheppard and Mills, 2003) and fear and anxiety scores in response to a thunderstorm recording (Landsberg et al., 2015). It also alleviates behavioral and neuroendocrine perioperative stress responses (Siracusa et al., 2010) and increases relaxation and reduces anxiety (but not aggression) in a veterinary clinic (Mills et al., 2006). It has also been shown to reduce stress-related behaviors (Gaultier et al., 2008) and fear of novel people (Gaultier et al., 2009) in recently adopted puppies. Furthermore, there is evidence that DAP reduces undesirable behaviors related to dogs’ separation from their owners, with a similar efficacy as using the psychotropic drug, clomipramine (Gaultier et al., 2005). In addition, it reduces separation-related anxiety signs during hospitalization (Kim et al., 2010), and reduces fear and anxiety in puppies during training and enhances socialization up to 1 year later (Denenberg and Landsberg, 2008). There are currently no known studies of the efficacy of DAP in enhancing “following” behaviors in response to human social cues or blood measures of OT or prolactin related to DAP use.

If both DAP and intranasally administered OT lead to increased levels of OT in the brain, exposure to both DAP and OT intranasal administration could have similar effects on consequent measures of blood neuro-hormone levels, and possibly on OCT performance. Hence, the aim of this study was to (i) compare the enhancing effects of DAP and intranasally administered OT on plasma levels of OT, and prolactin, and dogs’ OCT performance, against control and (ii) to explore the predictive power of: dog gender (as Oliva et al. (2015) demonstrated that male dogs perform better on the OCT after saline, but females respond more to the oxytocin intranasal treatment), origin (pet or foster dog), dog weight, home location (inside/outside – ambient temperature is known to influence levels of prolactin in the body, which may have flow on effects for the individual (see review, Alamer, 2011), point-following ability (spontaneous/non-spontaneous – refer to Materials and Methods section), plasma OT levels, serum prolactin levels, owner avoidant attachment scores, owner anxious attachment scores, EDED scores, and treatment, on OCT performance. Finally, the study aimed to identify differences in pet owner versus puppy carer attachment, as well as pet versus foster dog prolactin levels. It was hypothesized that, (i) plasma levels of OT will increase and serum levels of prolactin will decrease following DAP and OT exposure and that OCT performance will be enhanced by both OT and DAP compared to placebo. We also expected (ii) higher levels of plasma OT to be present in better performing dogs and better performing dogs to be more likely to be male and to be pets, while higher EDED scores and levels of prolactin to be present in poorer performing dogs. Lastly, we expected that foster owners would demonstrate greater avoidant attachment toward their dogs compared to pet owners and that foster dogs would have higher levels of serum prolactin compared to pet dogs.

## Materials and Methods

### Animals

A sample of 51 dogs and their owners or volunteer carers were recruited for the study. All dogs were more than 10 months old and recruited from either the assistance dogs association, Frédéric Gaillanne Foundation (FGF), or from owners that heard about the study via word of mouth, poster advertisements in pet shops, groomers or on the social media website, Facebook. Dogs who, at physical examination, were found to be pregnant, lactating, sensory impaired, or less than 2 kg were not included in the study. All entire females were tested on the OCT at least 2 months out of estrus. All dogs recruited from the FGF were in training and still living part time with the same human carer who had been assisting the FGF to raise them since they were between 8 and 10 weeks old. Henceforth these dogs shall be referred to as “foster dogs” and their volunteer carers as “puppy carers.” Dogs were to be excluded from the study if they showed signs of physical illness or extreme social phobia or aggression. The assessment of the dogs’ emotional status according to the EDED (refer to Materials section) scale did not reveal any emotional disorders which could have resulted in a non-inclusion. All dogs fell into the “normal” range, except for two pet dogs allocated to the DAP treatment group who fell into the “phobic” range. One foster dog was found to be pregnant at examination and was thus not included in the study. Two pet dogs displayed aggression in session 1 and were thus excluded. Two additional foster dogs changed puppy carers between sessions and were also excluded, thereby leaving a final sample of 46 dogs, 25 pets [17 Males (4 entire), 8 Females (2 entire)] and 21 foster dogs [9 Males (0 entire), 12 Females (2 entire)]. Tables 1 and 2 show details of the population involved in the study. The study was approved by the IRSEA Ethics Committee, approval number AFCE_201605_02.

**TABLE 1:** Study population descriptive data separated by treatment allocation group and origin.

	**Origin**		** Age (years)**		**Gender**		**Point-following ability**		**Location**			**Weight (kg)**		**Time owned/in care (years)**	
	**Pets**	**Fostered**	***M***	**SD**	**Male**	**Female**	**Spontaneous**	**Non- spontaneous**	**Inside**	**Outside**	**Both**	***M***	**SD**	***M***	**SD**
Oxytocin	8	6	3.5	2.9	8	6	5	9	6	2	6	25.9	10	2.9	2.3
Placebo	8	8	3.1	2.9	9	7	5	11	7	2	7	25.9	12.2	2.3	2.7
DAP	9	7	2.3	2.3	9	7	5	11	5	3	8	27.6	6.4	1.6	1.5
Pets	–	–	4.9	3.0	17	8	8	17	10	4	11	22.8	11.7	3.8	2.8
Fostered	–	–	1.1	0.2	9	12	7	14	8	3	10	30.9	3.7	0.9	0.1

**M* = mean, *SD* = standard deviation.*

**TABLE 2:** Breed differences between pet versus fostered dogs.

**Origin of dogs**			
**Pet dogs**		**Fostered dogs**	
**Breed**	***N***	**Breed**	***N***
Mixed	3	Bernese Mountain dog × Labrador (St Pierre)	14
Border Collie	3	Labrador	6
Welsh Corgi Cardigan	2	Labrador × Golden Retriever	1
Australian Shepherd	2		
German Shepherd	2		
Labrador	2		
Border Collie Cross	1		
Australian Shepherd × German Shepherd	1		
Bernese Mountain Dog	1		
Labrador × Boxer	1		
Malinois	1		
Samoyed	1		
Poodle	1		
Yorkshire Terrier	1		
Westie	1		
Chihuahua Cross	1		
Pinscher × Chihuahua	1		

**N* = sample size.*

### Materials

Twenty-four international units of OT (Sigma, St Quentin Fallavier, France) diluted in 0.2 ml of 0.09% saline, or 0.2 ml of 0.09% saline only (acting as a control) were administered to the nostrils of each dog, with a half-dose in each nostril. Treatments were delivered using a Mucosal Atomizer Device (Teleflex Medical SAS, La Pousaraque, France) connected to a 1 mL syringe. Treatments were prepared by a team member who did not take part in the experimental testing and were labeled as “A” or “B” to ensure that the researchers involved in the experiment were “blind” to the treatment conditions.

Adaptil^®^ collars impregnated with DAP or identical collars without impregnated DAP (placebo) provided by Ceva Santé Animale (Libourne, France), were fitted to the dogs’ neck in order to continuously expose dogs to DAP or placebo. Dogs were wearing the collar at least 1 day (24 h) before the OCT. Collars were also labeled as “A” or “B” by the same team member who did not take part in the experimental testing to ensure that the researchers involved in the experiment were “blind” to the treatment conditions. According to the combination of double-blinded intranasal and collar treatments that the dog received, it belonged to a treatment group called “A,” “B” or “C,” which the experimenters were completely blinded to as well, until after the data analysis was complete.

Two identical, opaque spaniel plastic bowls (19 cm base diameter, 11 cm rim diameter, 12 cm high, 8 cm deep) were used to conceal the food treats. Spaniel bowls were selected for their height and ability to conceal the treat from the dogs’ vision. Two additional and identical spaniel bowls were placed underneath the two testing bowls and treats identical to those used in the experiment were hidden in the space between them. This method was used by Udell et al. (2008a) and Oliva et al. (2015) to ensure that both bowls smelled of the treats and the dog was consequently not able to rely on olfaction when making its choice between the bowls. The treats used were pieces of poultry and vegetable flavored Frolic brand dry treats. Scores were marked by the experimenter using pen and paper.

The PAQ (Zilcha-Mano et al., 2011) and EDED scale (Pageat, 1995) were completed using pen and paper. Dogs who obtain an EDED score between 9 and 13 are classified as having a “normal emotional state,” dogs with a score of 14–16 are considered “phobic” and dogs scoring between 17 and 35 are considered “anxious.” Dogs who receive a score beyond 35 are classified as having a thymic or mood disorder (Pageat, 1995; Pageat and Fatjó, 2013).

### Procedure

The temporal unfolding of the overall procedure, comprising four steps (phone interview, session 1, session 2, and session 3), is described in Table 3. The different tasks described within the steps’ procedure are detailed hereunder.

**TABLE 3:** Temporal unfolding of the procedure and actions performed in each of its steps.

**Procedure steps**	**Time lapse**	**Actions**
Phone interview	D0	Phone contact with the dog owners/carers to check the inclusion criteria
		Appointment scheduling for session 1
Session 1 (S1)	D1	Physical examination at the Clinical Ethology and Animal Welfare Centre (CECBA) to confirm dog inclusion
		Explanatory statement and consent form signing
		EDED scale completion
		Stratification test
		Allocation in one treatment group
Session 2 (S2)	D2 = D1 or any day in between until at least 1 day before D3 [range of +1 to +107 days (*M* = 16.67, SD = 19.10)]	Blood drawing
		24–48 h before S3, fitting dogs with the collar (follow-up phone calls were made to ensure that this was done at the correct time)
Session 3 (S3)	D3 = D1 + 47 days on average [range of +1 to +232 days (*M* = 47.17, SD = 48.64)]	Food deprivation 6–8 h before the OCT to enhance motivation toward the treats
		Intranasal administration of one of the treatments (saline or OT)
		Blood drawing 15 min after treatment administration
		PAQ completion, dog free to explore the testing room
		OCT beginning 45 min after treatment administration: warm-up phase, testing sessions

*D = days.*

#### Stratification Test

The dogs participated in a “stratification test” to determine their classification as “spontaneous point followers” or “non-spontaneous point followers,” which would then inform their semi-random allocation to a particular treatment group. The setup of the stratification test consisted of three spots in a triangle shape on the floor, marked by small pieces of blue tape, 145 cm apart. The owner/puppy carer was asked to stand in the center of the triangle, connected to their dog by a leash. The owner/carer then randomly pointed five times at the spots, ensuring that each spot was pointed at once, without pointing at the same spot two times in a row. The behavioral veterinarian recorded how many times the dog correctly approached the pointed at spot. Dogs that followed their owner’s/puppy carer’s points correctly four or five times, out of the possible five were classified as “spontaneous point followers” while dogs that followed their owner’s/carer’s points three times or less were classified as “non-spontaneous point followers.”

Following the stratification test, and according to their classification (spontaneous vs. non-spontaneous), their origin (fostered dog vs. pet) and their sex, they were then allocated into one of three treatment groups (DAP active and placebo = DAP, DAP sham and placebo = control/placebo, DAP sham and OT = OT) in a pseudo-randomized and counterbalanced way. Point-following ability was included in the counterbalancing of treatment groups so as not to have one group containing dogs with more of a “spontaneous” point-following ability. This was decided in line with previous reports of dogs exhibiting a wide range of individual variability of OCTs.

#### Blood Drawing

Blood was taken in sessions 2 and 3 for subsequent measurements of basal and “treatment-induced” OT and prolactin levels respectively. Owners were asked to keep their dogs indoors the night before both sessions 2 and 3 to minimize the effect of ambient temperature on neurohormone secretion. Blood was drawn by a veterinarian from the jugular vein or from the cephalic vein, depending on the preference of the veterinarian for the dog, with the help of an operator. Most of the time, the dog was taken to a nearby room so that this was performed in the absence of the owner/puppy carer. Dogs received a treat immediately afterward in order to reduce stress responses. Up to 8 ml of blood was collected into pre-chilled pink EDTA-Aprotinin vacuum tubes (BD^®^ tubes, Elvetec, Pusignan, France) and 1 ml into red vacuum tubes with gel separator (Vacuette^®^ Greiner Bio-One, Alcyon, Paris, France), using either a 25G, 23G or 21G needles, depending on the size of the dog. The pink tubes were immediately transferred to an ice compartment where they remained until centrifugation while red tubes remained at room temperature for between 30 and 180 min. Samples were centrifuged at 1,600 × *g* for 15 min at 4°C. The plasma or serum was then transferred to a plastic tube and stored at −20°C.

#### Object Choice Task

##### Warm-up phase

The dog was first introduced to the bowls by the experimenter, which each contained one treat. The experimental set-up was similar to that of Virányi et al. (2008) and Oliva et al. (2015). The two spaniel bowls were placed 1.5 m apart and the experimenter knelt 30 cm behind the mid-point between the bowls. The dog sat or stood in between its owner and an operator, restrained by the operator by its collar, and faced the experimenter at a distance of 2.5 m. The owner/puppy carer, although present in the room at all times, did not participate in the task in any way, except in some cases to initiate participation in the task, as described in the scoring section below. The experimenter first got the dog’s attention by calling its name. The dog was then shown a treat before it was placed in one of the bowls in the dog’s full vision. The experimenter then said the release word “va” (the French word for “go,” or something equivalent if it was more familiar to the dog). The operator then released the dog and allowed it to approach one of the food bowls. If the dog approached the bowl containing the treat, it was allowed to eat the treat before both bowls were collected by the experimenter; if the dog approached the empty bowl or the experimenter, they immediately collected both bowls and the dog did not receive a treat. The warm-up phase consisted of four trials regardless of the dog’s performance, so long as the dog chose a bowl at least two out of the four trials. In cases where the dog did not choose a bowl at least two out of the four trials, the warm-up phase continued until two choices were made, or the dog was excluded. Dogs were excluded after 10 min failing to make two choices. After each trial, the experimenter stood up with both bowls and walked to the side of the room which was blocked from the dog’s vision by a barrier where they took another treat in their hand, while the operator fetched the dog and brought it back to the starting position. Once the dog was at the starting position, the experimenter returned and placed the bowls in their position on the floor and started the next trial.

##### Testing session

The experimental set-up was the same as in the warm-up phase. The testing session comprised two blocks of fifteen trials (10 where a pointing cue was provided and five control trials in which no cue to the treat’s whereabouts was provided). The control condition was used to verify that the dogs were not relying on scent to find the hidden food. Numerous studies have found that performance is at chance level (Hare et al., 2002; Soproni et al., 2002; Riedel et al., 2008; Udell et al., 2008a; Wobber et al., 2009; Machitella et al., 2017), or below chance level (Oliva et al., 2015) when a control condition is employed. Each block comprised, in sequence: three control trials, five trials with a momentary distal pointing cue, two control trials and then another five trials with a momentary distal pointing cue, in accordance with the sequence in Oliva et al. (2015). Having only 15 trials per block was strategically designed to keep the dog motivated. Furthermore, the dog was allowed approximately 5 min break between testing blocks to avoid burnout. Position of the correct bowl (left or right) was predetermined according to the same pseudo-randomized chart used in Oliva et al. (2015) that did not allow more than two consecutive trials where food could be obtained on the same side. As in the warm-up phase, after each trial, the experimenter stood up with both bowls and walked to the side of the room blocked from the dog’s vision by a barrier where they baited one of the bowls, while the operator fetched the dog and brought him/her back to the starting position. Once the dog was at the starting position, the experimenter returned and placed the bowls in their position on the floor and started the next trial.

##### Momentary distal point cue

The experimenter was kneeling, propped up on their toes, with their arms by their side. They got the dog’s attention and then rose their ipsilateral arm and pointed (using their index finger) toward the correct bowl for 1–2 s, keeping their head straight, before lowering their arm back down to their side and saying “va” (or an alternative release word more familiar to the dog). The approximate distance between the experimenter’s index finger and the rim of the baited bowl was 42 and 50 cm to the treat inside. The dog was then released and allowed to make a choice between the bowls.

##### Control condition

The kneeling experimenter, propped up on their toes, got the dog’s attention, kept their head straight for 1–2 s, then said “va” (or an alternative release word) before the dog was released by the operator and allowed to make a choice in the absence of any cue.

##### Scoring of the OCT

Scores were recorded as correct responses out of 10 trials per block (20 per test). If the dog did not move within 5 s of being released, the cue was given again by the experimenter. The dog may also have been prompted once by the owner if instructed to do so by the operator. If the dog did not approach a bowl within 5 s, the score for that trial was “no choice.” “No choices” were also recorded if the dog approached the experimenter instead of a bowl.

### Hormone Analysis

Prolactin was assayed in serum using the Prolactin canine ELISA kit (Demeditec, Kiel, Germany) following the manufacturer’s instructions. Plasma OT was assayed in the Oxytocin ELISA kit from Cayman Chemical (Arbor Inn, MA, United States) according to the manufacturer recommendations, including the plasma solid-phase extraction. Szeto et al. (2011), McCullough et al. (2013), and Christensen et al. (2014) have highlighted the crucial importance of carrying out this step, due to the fact that studies which have been published using unextracted samples have yielded levels far higher than extracted samples, leading to discrepancies in findings. Because of OT levels inferior to the kit’s sensitivity and in accordance with the manufacturer’s suggestion, 1.2 ml of plasma was first extracted on C18 columns (Hypersep 1 g, Thermo Fisher Scientific, Illkirch, France) followed by elution in 98% acetone. After drying the samples by vacuum centrifugation, they were resuspended in 0.6 ml assay buffer, hence allowing a two times concentration factor. The available plasma volumes of four samples in sessions 2 and 6 samples in session 3 turned out to be too small to be assayed using this method. The global procedure of extraction/concentration and assay was first internally validated on seven Quality Control samples of dog plasma from an external study to assess the precision and the accuracy of the whole method through the kit’s dynamic range: the mean precision was 12.0% and the mean recovery was 98.3%. Of note, some of the authors have recently published a validation of another Oxytocin ELISA kit from Enzo Life Sciences (Bienboire-Frosini et al., 2017) to assay OT in dog’s plasma. This kit was initially used in this study but unfortunately lead to too many results under the limit of detection. Therefore, we decided to use the Oxytocin ELISA kit from Cayman Chemical with a lower limit of detection, such as in MacLean et al. (2017).

### Statistical Analysis

Data analysis was realized thanks to SAS 9.4 software Copyright (©) 2002–2012 by SAS Institute Inc., Cary, NC, United States. Bilateral situation; the significance threshold was classically fixed at 5%.

#### Object Choice Task Performance

For the control conditions, correct attempts were totalled across the two blocks to give a score out of a possible 10 for each dog. Number of attempts made to the dog’s left bowl and the dog’s right bowl were also calculated. For the cued conditions, correct attempts for each of the testing blocks were combined to give a total raw score out of 20 for each dog, as well as a score for how many choices the dog made out of a possible 20. Then, a percentage was calculated for each dog as to the number of correct choices out of the total choices made. The same was done for the control conditions, whereby correct attempts for each block were combined to give a total raw score out of 10 for each dog. The data set was visually inspected for missing data and data entry accuracy. Two male pet dogs (1 entire, 1 neutered) were too scared to approach the experimenter/bowls in the warm-up phase of the OCT and so data pertaining to this part of the study could not be obtained in these cases. Furthermore, an additional 3 neutered male pet dogs were not motivated enough by the food (only by pets from the experimenter) to participate in the warm-up phase of the OCT and so OCT data could not be obtained from these dogs either. Hence the total sample size used in the analysis of the OCT was 41 dogs.

##### Validation of the experimental set-up

Validation of the experimental set-up was determined by firstly investigating side bias individually for each dog during the control and cued trials, based on the number of times a dog chose the right side and the number of times a dog chose the left side compared to the proportion 50% using a binomial test using the FREQ procedure. Secondly, validation of the experimental set-up was determined by analyzing whether dogs collectively performed the OCT above chance during the control trials i.e., in the absence of a cue (chance is 50%). Only dogs who made five or more bowl choices were included in this analysis, extrapolating the logic of including only dogs that made 10 out of 20 choices for the cut-off in the control trials explained below. Incidentally, after nine dogs were removed due to this cut-off, all remaining dogs made six choices or more. A one sample student *t*-test was realized using the TTEST procedure for this analysis.

##### Object choice task outcomes

To test for the effect of treatment group on OCT performance, two different outcomes were compared between the groups: (i) the number of “no choices” made and (ii) the percentage of correct choices made. Only dogs who chose 10 or more times (out of a possible 20) were included in the analysis pertaining to the second outcome. This was done because we believe a percentage calculated for a binary outcome (correct vs. incorrect), can be problematic because a certain amount of trials are needed to show if the performance is above chance level or not. For example, dogs who made only one or two choices could accidentally perform 100% correct or 100% incorrect, but this would not be a reliable reflection of their ability. Furthermore, using dogs who made less than 10 choices also makes them difficult to compare with previous studies which have generally used averages from 10 or more trials (Miklósi et al., 1998, 2005; Soproni et al., 2001; Udell et al., 2008b; Udell et al., 2010; Gácsi et al., 2009; Oliva et al., 2015). Hence, 7 pet dogs and 3 assistance dogs were excluded; 5 from the oxytocin treatment group, 1 from the placebo treatment group and 4 from the DAP treatment group. However, before comparing groups on the second outcome, we wanted to determine whether dogs in each treatment group (OT, placebo, DAP) and each recruitment group (pets and foster dogs) could perform the OCT above chance levels (50%). For this comparison, data analysis was performed by using a one sample Student *t*-test realized with the UNIVARIATE procedure. To compare performance between treatment groups, data analysis was performed using a one-way ANOVA by using the GLM procedure after testing that residuals were normal (verified with the UNIVARIATE procedure) and variances were homogeneous (verified with the GLM procedure). *Post hoc* multiple comparisons were carried out using the LSMEANS statement in PROC GLM using the Tukey-Kramer adjustment. For the data pertaining to the percentage of correct choices, the normality and the homoscedasticity of the data were verified, and a parametric independent samples ANOVA was then conducted. For the data pertaining to number of “no choices” made, normality and homoscedasticity was tested and found to be homogeneous between groups but not-normal. Hence, the non-parametric Kruskal–Wallis test was performed using the NPAR1WAY procedure.

#### Neurohormonal Parameters

##### Comparison of plasma oxytocin levels between sessions and treatment groups

To evaluate differences in plasma OT levels, plasma levels between sessions and treatment groups were compared. Conditions of normality and homogeneity of variances were verified with the UNIVARIATE and GLM procedures, respectively. Normality of data pertaining to plasma OT levels were found to be normal. Homoscedasticity was found to be verified. Given this, a General Linear Mixed Model was conducted using the MIXED procedure to evaluate the main and combined effects of treatment and session on OT levels.

##### Comparison of serum prolactin levels between sessions and treatment groups

Data pertaining to serum prolactin levels were found to be non normal. Other distributions were considered but were not found to be appropriate. Therefore, data was box-cox transformed but was still non normal after transformation. As such, only graphs can be presented to depict differences between sessions and treatment groups.

##### Comparison of serum prolactin levels according to origin

Average prolactin levels for each dog were calculated by calculating the mean serum prolactin value obtained from sessions 2 and 3. Data analysis was carried out in the following way: assumption of normality was verified using the UNIVARIATE procedure and homogeneity of variances using the TTEST procedure. Prolactin levels in pet and foster dogs were found to be skewed toward the lower range. An atypical value was also obtained in the foster dog sample. Variances were homogeneous, but due to the non normal distribution of the data a non-parametric Wilcoxon two-sample test was done, using the NPAR1WAY procedure, to compare levels between groups. The analysis was performed with and without the atypical value and it was not found to influence the results and hence, to maintain the integrity of the data the results including the atypical value is presented in the “Results” section.

#### Predicting Object Choice Task Performance

Backward deletion multiple regressions were conducted to identify significant predictors of OCT performance on the two outcome variables: (i) the number of “no choices” made and (ii) the percentage of correct choices made. For the same reasons described above in relation to the second outcome, only dogs who chose a bowl 10 or more times were included in this multiple regression analysis. Multiple regressions were realized for qualitative explicative variables: dog gender (female entire/female spayed/male entire/male neutered), origin (pet dog/fostered dog), weight, home location (inside/outside/both), point-following ability (spontaneous/non-spontaneous), session 3 plasma OT levels, session 3 serum prolactin levels, owner avoidant attachment scores, owner anxious attachment scores, EDED scores, and treatment group (OT/DAP/placebo). Homogeneity of variances was verified using the HOVTEST = LEVENE option in the MEANS statement of GLM procedure. Normality was verified on complete model residuals using the UNIVARIATE procedure and the complete model was done using the GLM procedure. After realizing the complete model, simplification of this was done using backward selection method. The aim of simplification was to find the best model for the data that maximizes the *r*^2^ of the model. The GLMSELECT procedure with the SELECTION = BACKWARD option on the MODEL statement was used for this purpose. The selected model was then studied using the GLM procedure. Data pertaining to the percentage of correct choices was found to be normal and homogeneity was also found between groups.

#### Comparison of Human Attachment

Avoidant and anxious attachment to dogs were compared for pet owners and puppy carers. Data analysis was carried out in the following way: conditions of normality and homogeneity of variances was verified (with respectively the UNIVARIATE procedure and the TTEST procedure). Normality of data pertaining to owner/carer avoidant attachment scores toward their pet dogs and their foster dogs was found to be skewed toward the lower end in pet owners. Normality of data pertaining to owner/carer anxious attachment scores toward their pet dogs and their foster dogs was found to be skewed toward the lower end in both groups. Variances between groups were homogenous for both sub-scales but due to the non normal distribution of scores non-parametric Wilcoxon two-sample tests were employed to identify group differences.

## Results

### Object Choice Task Performance

#### Validation of the Experimental Set-Up

Of the 41 dogs that were analyzed in the control trials, nine demonstrated a significant side bias, representing 21.9% of the population. Seven dogs (17% of the population) chose the bowl to their left side significantly more than the bowl to their right and two dogs (4.9% of the population) chose the bowl to their right side significantly more than the bowl to their left (*p* < 0.05). Of the 9 dogs, 6 were fostered dogs and 3 were pets, with 5/9 belonging to the OT group, 3/9 belonging to the DAP group and 1/9 belonging to the control group. Eight out of nine of these dogs demonstrated a bias for the side where they experienced their first food reward.

Of the 39 dogs that were analyzed in the cued trials (two dogs did not make any choices in the cued trials), 10 demonstrated a significant side bias, representing 25.6% of the population. Seven dogs (17.9% of the population) chose the bowl to their left side significantly more than the bowl to their right and three dogs (7.7% of the population) chose the bowl to their right side significantly more than the bowl to their left (*p* < 0.05). Of the 10 dogs, 8 were fostered dogs and 2 were pets, with 1/10 belonging to the OT group, 5/10 belonging to the DAP group and 4/10 belonging to the control group. Seven out of ten of these dogs demonstrated a bias for the side where they experienced their first food reward. Interestingly, only three dogs with a left side bias in the control trials also demonstrated the same left side bias in the cued trials, and only one dog with a right side bias in the control trials also demonstrated the same right side bias in the cued trials.

One sample student’s *t*-test revealed the percentage of correct choices in the control trials, where no cue was given to indicate the location of the hidden treat, was not significantly different from chance (50%) (*M* = 55.72, 95% CI [49.85; 61.59], SD = 16.27; *t* = 1.99, *p* = 0.056), indicating that the dogs were performing at chance levels in the absence of a cue.

#### Outcome 1: Number of “No Choices” Made

Table 4 shows the average number of times dogs in each treatment group did not make a choice following a given cue and in the absence of any cues (control trials).

**TABLE 4:** Mean and median number of times dogs in each treatment group did not make a choice in the cued trials (out of 20) and in the control trials (out of 10).

**Type of trial**	**Group**	***N***	***M***	**SD**	**Lower 95% CI**	**Upper 95% CI**	**Median**	**Lower quartile**	**Upper quartile**	**Min**	**Max**
Cued	Oxytocin	13	7.69	9.22	2.12	13.27	2	0	18	0	20
	Placebo	13	2.62	5.69	−0.83	6.06	0	0	2	0	20
	DAP	15	5.27	6.88	1.46	9.08	1	0	1.46	0	18
Control	Oxytocin	13	3.23	3.49	1.12	5.34	1	0	7	0	8
	Placebo	13	1.54	2.47	0.05	3.03	1	0	2	0	9
	DAP	15	2.93	3.08	1.23	4.64	2	1	4	0	9

**N* = sample size, *M* = mean, SD = standard deviation, CI = confidence interval.*

A Kruskal–Wallis test revealed a non-significant effect of treatment group on number of “no choices” made for trials where a cue was provided, *X*^2^ (2, *N* = 41) = 1.96, *p* = 0.374.

#### Outcome 2: Percentage of Correct Choices Made

Table 5 shows the percentage of correct choices made by the whole population of dogs in the sample.

**TABLE 5:** Mean percentage of correct choices made by all dogs in each treatment group.

**Group**	***N***	***M* (%)**	**SD (%)**	**Lower 95% CI**	**Upper 95% CI**	**Min (%)**	**Max (%)**
Oxytocin	12	58.87	38.99	34.09	83.64	0	100
Placebo	13	57.05	22.74	43.31	70.79	0	90
DAP	15	63.15	16.28	54.14	72.17	33.33	94.12

**N* = sample size, *M* = mean, SD = standard deviation, CI = confidence interval.*

On the whole population, the highest mean percentage of correct choices and the lowest standard deviation are interestingly both obtained for the DAP group. Indeed, the standard deviations are quite large, particularly for the OT group. This is not surprising given that some of these dogs only chose a very small number of times, or in two cases, made “no choices” at all (refer to scores in Table 4) thereby obtaining a percentage of correctness that was based on 0–2 trials only (see Table 6 for raw scores). Therefore, based on the logic explained in the “Statistical Analysis” section, it was decided to only include dogs that chose 10 times or more for inclusion in the ANOVAs in the following section. Tables 6 and 7 show the pattern of performance of the dogs that chose at least once but less than 10 times and were therefore excluded from the ANOVAs.

**TABLE 6:** Raw correct scores (out of total choices made by dog) for dogs that chose at least once but less than 10 times in the object choice task according to treatment group.

	**Treatment**	
	**Oxytocin**	**DAP**
	0/1^∗^ 0/2† 1/2^∗^ 0/1^∗^	1/2^∗^ 1/3† 3/5† 6/9^∗^
Average number of attempts	1.5	4.75

*^∗^Pet dog, ^†^fostered dog.*

**TABLE 7:** Total number of dogs who made less than the total number of attempts set in the test (i.e., <30), the percentage of these dogs that started the OCT with a “No Choice”, the total number of stopping events after successes and failures, and the percentage of these dogs who approached the experimenter at least once during their “Stopping Event.”

**Group**	***N***	**% started with no choice**	**Average number of choices not made in control trials (out of 10)**	**Average number of choices not made in cued trials (out of 20)**	**Total stopping events after success**	**Total stopping events after failure**	**Average % of times dogs approached experimenter as their “no choice”**
Oxytocin	7	57.14	5.71	14.29	5	12	22.39
Placebo	5	40	3.20	7	3	8	36.55
DAP	9	44.44	4.44	8.78	9	16	39.26
Total	21	47.62	4.57	10.19	17	36	33.70

**N* = sample size.*

What is immediately striking from Table 6 is that none of the numerators are equal to the denominators, meaning that all these eight dogs experienced failure at some point during the task. The dogs in the OT group comprised 3 pet dogs and 1 foster dog while the DAP group comprised 2 pet dogs and 2 foster dogs, so origin does not appear to influence why these dogs made so few choices. To further investigate why some dogs might have stopped making choices, we looked at the rates of success or failure of the previous attempt before the dogs decided to stop. This was done for all dogs who started the OCT and either stopped completely (did not make any further attempts) or stopped and then started again, including both the control trials (10) and the cued (i.e., pointing) trials (20).

As can be seen from Table 7, almost half the dogs that made less than 30 attempts on the OCT started the task (which began with a control, i.e., un-cued trial) by not making a choice. Furthermore, dogs in all treatment groups stopped participating (i.e., did not participate in at least the next trial) more often after a failed attempt than after a successful one. This may suggest that their prior success (or failure) is influencing their decision to make a future attempt. Non-participation included anything that did not involve making a selection between the two bowls, so non-participation also included approaching the experimenter. While not considered participation in the task *per se*, this could still have been considered by the dogs as a potential strategy to obtain food. To investigate whether dogs were using this strategy more or less in a particular treatment group, percentages were calculated indicating how often this approach was used during a “stopping event” (refer to Table 7). By eye-balling these percentages, it appears that the OT group used this non-rewarding technique less than the other two groups.

##### Compared to chance

One samples *t*-tests were used to compare whether dogs in each treatment group and each recruitment group could perform the OCT significantly better than chance. As explained above, only dogs who chose 10 or more times were included in this analysis. Percentage of correct choices made were compared to the chance level of 50%. Dogs in all groups were found to perform the OCT above chance, as can be seen in Table 8.

**TABLE 8:** Mean percentage of correct choices made by dogs (that chose 10 or more times) in each treatment and recruitment group compared to chance (50%).

**Group**	***N***	***M* (%)**	**SD (%)**	**Lower 95% CI**	**Upper 95% CI**	**Min (%)**	**Max (%)**	***t* statistic**	***p*-value**
Oxytocin	8	82.05	16.70	68.09	96.01	50	100	5.43	0.001
Placebo	12	61.80	15.60	51.89	71.71	35	90	2.62	0.024
DAP	11	67.03	15.69	56.49	77.56	47.37	94.12	3.60	0.005
Pet	13	75.32	17.49	64.75	85.88	50	100	5.22	0.0002
Fostered	18	64.23	16.30	56.13	72.34	35	90	3.70	0.0018

**N* = sample size, *M* = mean, SD = standard deviation, CI = confidence interval.*

##### Comparison between treatment groups

An independent samples ANOVA revealed that there was a significant effect of treatment group (DF = 2, *F* = 4, *p* = 0.030). *Post hoc* multiple comparisons revealed that dogs that received OT chose the correct bowl significantly more than dogs in the control condition (Tukey, *p* = 0.0248). No other significant differences were found between groups.

### Neurohormonal Parameters

#### Comparison of Plasma Oxytocin Levels Between Sessions and Treatment Groups

Mean levels of plasma OT according to session (2 or 3) and treatment (OT, DAP or saline) can be seen in Table 9, as well as mean levels of plasma OT according to session only (including a combination of dogs from each treatment group). A mixed model ANOVA revealed a significant main effect of session (DF = 1, *F* = 5.36, *p* = 0.025), an insignificant main effect of treatment DF = 2, *F* = 0.92, *p* = 0.408) and an insignificant session × treatment interaction DF = 2, *F* = 2.13, *p* = 0.130). These findings indicate that plasma OT levels were significantly higher in session 2 than in session 3 in the whole dog population, regardless of the treatment given to dogs.

**TABLE 9:** Mean concentration of plasma oxytocin (pg/ml) in each session and in each treatment group separated by session.

	***N***	***M***	**SD**	**Lower 95% CI**	**Upper 95% CI**	**Min**	**Max**
**Session**							
2	40	29.27^∗^	14.07	24.77	33.77	5.43	66.54
3	38	22.71^∗^	13.29	18.34	27.08	5.09	56.60
**Group and session**							
Oxytocin S2	12	30.64	18.73	18.74	42.54	5.43	66.54
Oxytocin S3	12	27.17	16.55	16.66	37.68	5.09	56.60
Placebo S2	12	23.45	8.38	18.12	28.77	12.66	39.89
Placebo S3	11	21.44	13.57	12.32	30.56	7.89	50.23
DAP S2	16	32.61	12.86	25.76	39.46	14.20	53.56
DAP S3	15	20.08	9.71	14.70	25.46	8.26	35.31

*S = session, *N* = sample size, *M* = mean, SD = standard deviation, CI = confidence interval. ^∗^*p* = 0.025.*

#### Comparison of Mean Serum Prolactin Levels Between Sessions and Treatment Groups

**FIGURE 1:**
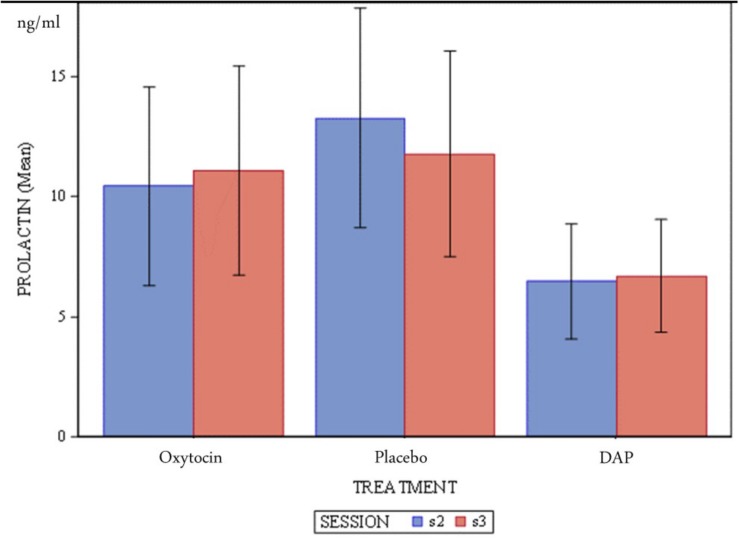
Mean concentration (ng/ml) of dog serum prolactin and standard error according to treatments and sessions.

Mean concentrations of serum prolactin between sessions and between treatments are shown in Figure 1. Visual inspection of Figure 1 suggests no differences between sessions, or treatment groups as the error bars all overlap.

#### Comparison of Serum Prolactin Levels According to Origin

Levels of serum prolactin for session 2 and session 3 were averaged for each animal independently. The median of these values were then calculated for pet dogs and foster dogs separately, and are presented in Table 10.

**TABLE 10:** Serum prolactin levels (ng/ml) in pet dogs versus foster dogs.

	***N***	***M***	**SD**	**Lower 95% CI**	**Upper 95% CI**	**Median**	**Lower quartile**	**Upper quartile**	**Min**	**Max**
Pet dogs	25	6.05	8.84	2.31	9.78	2.17^∗^	1.225	8.721	0.20	40.47
Foster dogs	21	14.73	17.43	6.79	22.67	8.39^∗^	3.214	18.810	0.20	68.81

**N* = sample size, *M* = mean, SD = standard deviation. ^∗^*p* < 0.025.*

A two-sample Wilcoxon Test showed that foster dogs had significantly higher levels of serum prolactin compared to pet dogs, *Z* (*N* = 46) = 2.27, *p* = 0.024.

### Predicting Object Choice Task Performance

Predictors of OCT performance were evaluated separately for the two outcome variables: (i) total “no choices” and (ii) percentage of correct choices made. Backward deletion multiple regressions were conducted for the two separate outcomes variables using the following predictor variables: dog gender (female entire/female spayed/male entire/male neutered), origin (pet dog/fostered dog), weight, home location (inside/outside/both), point-following ability (spontaneous/non-spontaneous), session 3 plasma OT levels, session 3 serum prolactin levels, owner avoidant attachment scores, owner anxious attachment scores, EDED scores, and treatment group (OT/DAP/placebo). Means and standard deviations for the categorical variables “gender,” “origin,” “home location,” and “point-following ability” included in the analyses are shown in Table 11. Refer to Table 8 for this information pertaining to treatment group.

**TABLE 11:** Means and standard deviations for the categorical variables “Gender,” “Origin,” “Home Location,” and “Point-Following Ability” included in the multiple regression analyses.

	**Total “no choice”**			**% Correct**		
	***N***	***M***	**SD**	***N***	***M***	**SD**
**Gender**						
Female entire	4	3.50	3.32	4	63.75	16.19
Female spayed	16	4.31	7.37	12	66.41	17.30
Male entire	3	6	10.39	2	57.50	10.61
Male neutered	18	6.22	8.16	13	74.49	18.48
**Origin**						
Pet	20	7.80	8.20	13	75.32	17.49
Fostered	21	2.71	5.91	18	64.23	16.30
**Home location**						
Inside	15	4.73	7.92	11	65.19	19.42
Outside	21	5.86	7.60	16	69.45	16.27
Both	5	3.80	6.38	4	76.75	17.99
**Point-following ability**						
Spontaneous	13	5	7.68	10	75.62	18.10
Non-spontaneous	28	5.29	7.53	21	65.67	16.58

**N* = sample size, *M* = mean, SD = standard deviation.*

For total “no choices”, the model containing all the predictors was non-significant. Variables were removed from the model following a backward selection in the following steps: (i) gender, (ii) home location, (iii) treatment group, (iv) avoidant attachment scores, (v) weight, (vi) session 3 plasma OT levels, (vii) session 3 serum prolactin levels, (viii) anxious attachment scores, (ix) EDED scores, and (x) point-following ability. Step 10 resulted in the greatest improvement of the model and reached significance, DF = 1, *F* = 11.93, *p* = 0.0015, *r*^2^ = 0.27, with “origin” explaining 27% of the variance in no choice scores. Regression coefficients for the significant model can be found in Table 12.

**TABLE 12:** Standardized and unstandardized regression coefficients for each predictor in the significant model of the backward deletion multiple regression for “no choice” outcome.

**Parameter**	***B***	**β**	**SE**
Intercept	8.71	0	1.55
Fostered	−7.48^∗^	−0.15^∗^	2.17
Pet	0	0	–

**B* = standardized regression coefficient, β = unstandardized regression coefficient, SE = standard error. ^∗^*p* = 0.0015, *r*^2^ = 0.27.*

Because the significant variable left in the model was categorical, an independent samples ANOVA was then run to further study the selected model coming from the backward selection procedure, (i.e., using only the “origin” variable which was the significant predictor for “no choice” outcomes). The ANOVA revealed that pet dogs were significantly more likely not to make a choice than foster dogs, DF = 1, *F* = 5.23, *p* = 0.028.

For percentage of correct choices made, the model containing all the predictors was non-significant. Variables were removed from the model following a backward selection in the following steps: (i) gender, (ii) home location, (iii) anxious attachment scores, (iv) session 3 plasma oxytocin, (v) EDED scores, (vi) avoidant attachment scores, (vii) weight, (viii) session 3 serum prolactin, and (ix) origin. Step 9 resulted in the greatest improvement of the model and reached significance, DF = 3, *F* = 3.89, *p* = 0.022, *r*^2^ = 0.34, with “point-following ability” and “treatment” explaining 34% of the variance in percentage correct scores. Regression coefficients for the significant model can be found in Table 13.

**TABLE 13:** Standardized and unstandardized regression coefficients for each predictor in the significant model of the backward deletion multiple regression for percentage correct outcome.

**Parameter**	**B**	**β**	**SE**
Intercept	75.88	0	5.90
Spontaneous	0	0	–
Non-spontaneous	−13.91^∗^	−0.40^∗^	6
Oxytocin	13.54^†^	0.35^†^	7.24
Placebo	–7.42	–0.21	6.73
DAP	0	0	–

**B* = standardized regression coefficient, β = unstandardized regression coefficient, SE = standard error. ^∗^*p* = 0.03, ^†^*p* = 0.07, *r*^2^ = 0.34.*

Because the significant variables left in the model were categorical, a two-factor ANOVA was then run to further study the selected model coming from the backward selection procedure, (i.e., using only “treatment group” and “point-following ability” as independent variables). In line with the previously presented independent samples ANOVA, the two-factor ANOVA revealed that percentage of correct choices made was significantly different according to “treatment group,” DF = 2, *F* = 4.86, *p* = 0.016, and *post hoc* multiple comparisons revealed that dogs who were in the “OT” group were more likely to choose correctly than dogs in the “placebo” group (Tukey, *p* = 0.014). A trend was also observed for “point following ability” whereby spontaneous dogs were more likely to choose correctly than non-spontaneous dogs, DF = 1, *F* = 3.82, *p* = 0.061.

### Comparison of Human Attachment

The range, and median scores for pet owner and puppy carer anxious and avoidant attachment scores can be seen in Table 14.

**TABLE 14:** Avoidant and anxious attachment scores in pet owners and puppy carers.

	***N***	***M***	**SD**	**Lower 95% CI**	**Upper 95% CI**	**Median**	**Lower quartile**	**Upper quartile**	**Min**	**Max**
Pet owner avoidant	25	1.57	0.61	1.32	1.82	1.20^∗^	1.20	1.70	1	3.40
Puppy carer avoidant	21	2.08	0.67	1.77	2.38	1.90^∗^	1.60	2.30	1.20	3.60
Pet owner anxious	25	2.34	0.99	1.93	2.74	2	1.60	2.70	1.20	4.80
Puppy carer anxious	21	2.42	0.92	2	2.84	2.20	1.60	3.30	1.40	4

**N* = sample size, *M* = mean, SD = standard deviation. ^∗^*p* < 0.0025.*

For avoidant attachment scores, a two-sample Wilcoxon Test showed that puppy carers were significantly more avoidantly attached than pet owners, *Z* (*N* = 46) = 3.03, *p* = 0.0024.

For anxious attachment scores, no significant differences were observed between groups analyzed with a two-sample Wilcoxon Test, *Z* (*N* = 46) = 0.30, *p* = 0.77.

## Discussion

This study aimed to further previous findings demonstrating that intranasal OT enhances dogs’ performance on an OCT (Oliva et al., 2015; Macchitella et al., 2017) by comparing the effects of OT and DAP on (i) number of choices made on an OCT and (ii) correctness of choices made on an OCT, when compared to placebo. The study also aimed to investigate the effects of dog gender (female entire/female spayed/male entire/male neutered), origin (pet dog/fostered dog), weight, home location (inside/outside/both), point-following ability (spontaneous/non-spontaneous), session 3 plasma OT levels, session 3 serum prolactin levels, owner avoidant attachment scores, owner anxious attachment scores, EDED scores, and treatment group (OT/DAP/placebo), on OCT performance. Finally, the study aimed to identify differences in pet owner versus puppy carer attachment, as well as pet versus foster dog prolactin levels.

### OCT Performance Compared to Chance

In line with previous findings (Oliva et al., 2015), all dogs that made more than 10 choices on the OCT were able to perform above chance. However, the difference in scoring between the two studies meant that more dogs in the current study made less than 10 choices. Indeed, when a dog did not make a choice in Oliva et al.’s study it was given a test of motivation, which involved two pre-training trials (one to each side). If the dog chose a bowl during this test of motivation, it was deemed to be motivated and thus an assumption was made that the previous “no choice” outcome was due to the dog not understanding the task and so was given a score of “incorrect choice” for that trial. In contrast, in the current study, the task purposely continued without a test of motivation, and a score of “no choice” was given to that trial. Another important difference in the study by Oliva et al. is that only two dogs did not pass the initial pre-training, in which the dog had to select the correct bowl four times in a row after being shown the treats being placed into the correct bowl. Inspection of Oliva et al.’s (unpublished) raw data revealed that, surprisingly, 42% of the 67 dogs who completed the initial pre-training needed more than four attempts to complete the initial pre-training, with 15% needing 10 or more trials. Furthermore, pre-training was repeated before each block, of which there were four per testing session. While the number of dogs needing more than four attempts per session reduced over the blocks within each session, the number of dogs requiring more than four attempts for the initial pre-training before session two rose back up to 52%, however only 3 out of 63 dogs who completed session 2 needed 10 or more attempts. Therefore, it is fair to say that the dogs in Oliva et al.’s study received a lot more pre-training compared to the dogs in the current study which were given four trials only, regardless of the dog’s performance, so long as the dog chose a bowl at least two out of the four trials. The reduced pre-training in the current study was a purposeful attempt to reduce the probability of dogs learning how to perform the task within the sessions, as was observed in Oliva et al. (2015). Still, the reduced pre-training in the current study did not appear to affect dogs’ ability to perform above chance, in dogs that were willing to make more than 10 choices. It may, however, have reduced the number of dogs willing to make 10 choices.

### The Influence of Oxytocin Versus DAP on OCT Performance

The hypothesis that OCT performance would be enhanced by both OT and DAP compared to placebo was only partially supported as the percentage of correct choices made in the OCT was enhanced by OT compared to placebo, but not DAP compared to placebo, in the sub-population of dogs who made at least 10 choices. These performance enhancing effects of OT are in line with previous studies (Oliva et al., 2015; Macchitella et al., 2017). In the current study we investigated performance in two ways (i) number of choices not made (based on the whole sample) and (ii) percentage of correct choices out of those made (based on the sample of dogs that made 10 or more choices, i.e., the “best choosers”). Our findings suggest that OT increases the percentage of correct choices made in the “best choosers” sub-population but not the number of choices made. This is an important finding, as previously it has been put forward that perhaps dogs are performing better due to a decrease in anxiety when performing the task (Oliva et al., 2015), however, the current findings do not suggest a willingness to “have a go” is the reason why dogs are performing better, as OT did not increase number of choices made. In fact, for the eight dogs that made at least one but less than 10 attempts when given the pointing cue, the four dogs belonging to the OT group demonstrated very low rates of choosing (1.5 choices on average) compared to the other four dogs belonging to the DAP group (who chose 4.75 times on average) (refer to Table 6). To investigate why this might be we looked at the sub-group of dogs (*N* = 21) who made less than 30 attempts (on both the control and cued trials) which revealed that dogs in all groups were more likely to stop performing the OCT (i.e., not participate in at least the following trial) after a failure than after a success (refer to Table 7). Hence, the very low number of attempts (<10) seen in the OT group may suggest that this group were more influenced by prior losses than the dogs in the DAP group. The reverse of this (i.e., being influenced by prior success) may have also been taking place in dogs who demonstrated a side bias, with the majority of these dogs developing a bias to the side where they first experienced a food reward. Interestingly, dogs in the OT group made up the majority of these dogs in the control trials at 56%, but reduced to the minority group in the cued trials at 10%, which may suggest that these dogs employed different techniques on the OCT, depending on whether a cue was offered or not. The OT group also contained the largest percentage of dogs who started the OCT (which always commenced with a control/no cue condition) by not choosing. It is possible that these dogs realized from the very beginning that they did not understand the task, or were being tricked that they had enough information to complete it correctly, and therefore made a beneficial decision to not perform – after all, if they don’t perform, they can’t be wrong. They also used the non-reinforcing strategy of approaching the experimenter (which resulted in the experimenter immediately picking up the bowls and walking away), less times than the other two groups. This may reflect a greater understanding in these dogs that this strategy would not result in a reward, food or otherwise, when performing this task, despite the fact that they might use this strategy to obtain food or attention from humans outside of the task. DAP has been shown to have effects as an emotional modulator in dogs (Sheppard and Mills, 2003; Gaultier et al., 2005, 2008; Tod et al., 2005; Mills et al., 2006; Denenberg and Landsberg, 2008; Kim et al., 2010; Siracusa et al., 2010; Landsberg et al., 2015). In this study, it did not significantly increase number of “no choices” made nor OCT performance in the “best choosers” population. However, it is noteworthy that on the whole population descriptive data, the DAP group displayed the lowest standard deviations/variability, suggesting a greater homogeneity in OCT performance for this group. This could be related to the general ability of appeasing pheromones in modulating/smoothing the cognitive-emotional responses among individuals, particularly in the context of cognitive tasks, as already described in horses treated with EAP (Equine Appeasing Pheromone, the homologous specific appeasing pheromone) by Mengoli et al. (2014). It could be possible that the “best choosers” dogs were already in this kind of balanced cognitive-emotional state, hence making us unable to conclude about DAP effects in this population.

An alternative explanation for the reduced choosing behavior observed in dogs treated with OT is that the putative “relaxing” effect of OT (Uvnas-Moberg and Petersson, 2005) may have caused them not to care to participate in the OCT since they were already in a kind of “rewarded” mental state of well-being and so did not need to work further to get a reward (refer to Table 7). It is also possible that the anorexigenic effects of OT impacted their motivation to perform a task where their efforts are rewarded by food (see reviews, Olszewski et al., 2010; Onaka et al., 2012; Spetter and Hallschmid, 2017).

### The Influence of Intranasal Oxytocin on Neurohormonal Parameters

The hypothesis that plasma levels of OT and serum prolactin will change following DAP and OT exposure was not supported. Treatment was not found to have any effect on plasma OT or serum prolactin levels. However, dogs in the OT treatment group demonstrated enhanced cognitive ability and so in relation to the modality of intranasal OT, our findings suggest that OT delivered intranasally reaches the brain directly, and not by a peripheral mechanism, as has been postulated (Lee et al., 2018). These findings are also discrepant with previous findings in dogs where an increase in levels of OT has been reported 15 min after intranasal application (Romero et al., 2014; Temesi et al., 2017). There are several reasons why our findings are discrepant with Romero et al. (2014). First, in their study 40 IU of intranasal OT was applied, vs. 24 IU used in the current study. As this is nearly double the amount, this could go some way to explaining the differences in findings between our study and theirs. Second, the number of dogs in their study was only five, compared to the 12 dogs that we were able to analyse in the OT treatment group. In addition to this larger sample size, we also observed a high rate of variability in our population, as evidenced in Table 9, which could have “hidden” any possible difference between the sessions and/or groups. Such wide variability in OT levels in plasma is not uncommon after intranasal administration and has also been reported in monkeys for instance (Lee et al., 2018). Indeed, a variability of approximately 50% of mean basal plasma OT concentrations has been previously reported within populations from several species, regardless of exogenous OT administration (Bienboire-Frosini et al., 2017). Crockford et al. (2014) precisely described and explicated the general variability of the oxytocinergic system within species. Third, the discrepancies could be due to differences in measurement procedures. For example, Romero et al. used radioimmunoassay (RIA) to assay OT in plasma, whereby the current study used EIA. With regards to Temesi et al. (2017) again, a small number of only six dogs was analyzed, and their methods to measure OT also differed from ours in an important way. For instance, they assayed OT in serum, not in plasma, and used a different ELISA kit, which has not been validated for use in dogs, to the best of our knowledge. Furthermore, the levels of OT they found after intranasal application (of 12 IU) is much higher than the one observed by Romero et al. with 40 IU intranasal application. Still, our lack of an OT increase in the blood is also inconsistent with findings from previous studies in rodents (Neumann et al., 2013), but may be explained by the fact that these changes take longer than 15 min to reach the blood. Indeed, Neumann et al. (2013) only observed a significant increase in blood 70 min following intranasal administration. This suggests that the OT first reaches the brain where it has measurable behavioral consequences and this may result in a downstream increase in the OT in the blood after 15 min. Conversely, other studies in primates and humans have showed that plasma OT could peak at 10–15 min after the intranasal administration (Striepens et al., 2013; Dal Monte et al., 2014). Future studies would need to be conducted to investigate this in dogs, using large samples and validated methods to measure OT in blood. Similar effects may also be observed in serum prolactin and future studies should take additional blood samples at various times to attempt to capture this.

Interestingly, in the current study, plasma OT levels significantly decreased from session 2 to session 3 in the whole population, with no influence of treatment, refer to Table 9. We can speculate that this decrease in OT between sessions reflects the dogs being less stressed/aroused during session 3. Indeed, previous authors showed that OT release in brain and plasma could be related to acute stress events (Wotjak et al., 1998; Onaka et al., 2012; Noller et al., 2013). Despite the veterinarians’ attempts to reduce the stressfulness of the first blood sampling, it is possible that this handling could still trigger an increase in endogenous plasma OT levels to dampen the HPA axis activation, as already observed in mini-pigs (Marcet Rius et al., 2018) and beef heifers (Wagner et al., 2019). In session 3, the dogs would have been more familiar with the veterinarians and the testing location and therefore may have felt more comfortable with the environment. The opposite possibility is also true that they may have formed a negative association with the veterinarians due to the blood sampling. However, for the dogs that received OT intranasally in session 3, this may have acted as an anxiolytic as it has been successfully demonstrated in humans (Heinrichs et al., 2003; de Oliveira et al., 2012). Similarly, for the dogs that received DAP, this too may have acted as an emotional modulator as has been previously demonstrated (Sheppard and Mills, 2003; Gaultier et al., 2005, 2008; Tod et al., 2005; Mills et al., 2006; Denenberg and Landsberg, 2008; Kim et al., 2010; Siracusa et al., 2010; Landsberg et al., 2015). Alternative methods have shown promise in more accurately measuring total (bound and unbound) levels of OT, the bound fraction of which needs to be unbound before being measured (Brandtzaeg et al., 2016). Future studies may consider the use of these methods in assessing whether bound levels of OT have an effect on OCT performance in dogs.

### Predicting OCT Performance

Contrary to our expectations, plasma OT levels did not differ in better performing dogs. In addition to our expectation that dogs with higher levels of plasma OT levels would perform better on the OCT, we also expected better performing dogs to be more likely to be male and to be pets, while higher levels of prolactin and EDED scores to be present in the poorer performing dogs. Our findings that gender did not predict performance are in contrast to Oliva et al.’s (2015) findings of an enhanced performance in male dogs compared to female dogs following intranasal saline administration, however, they are in line with their findings that gender no longer acted as a predictor following intranasal oxytocin administration. This may be explained by the significant treatment effect observed in our study. Interestingly, foster dogs were more likely to make a choice than pet dogs, but they were no more likely than pet dogs to choose correctly, refer to Tables 12, 13. This highlights an important point that just because the dog is performing, does not indicate that it has a better *understanding* of *how* to perform than a dog that chooses not to. Furthermore, foster dogs’ increased choosing behavior may not reflect greater willingness to have a go, but may be more reflective of their training and familiarity with following orders, compared to pet dogs. Alternatively, this could reflect a change in the wiring of these dogs’ brains, due to their particular upbringing. Previous studies have identified individual differences in OCT performance (Miklósi et al., 1998; Agnetta et al., 2000; Hare et al., 2002; Udell et al., 2008a, b; Udell et al., 2010; Virányi et al., 2008; Wobber et al., 2009; Oliva et al., 2016b). Oliva et al. (2016b) investigated whether this could have been due to differences in the OT receptor gene but were not able to demonstrate an association. Findings from the current study suggest that a dogs’ early social and learning experiences may impact their ability to use human social gestures effectively, and this may be more influential than their genetic blueprint.

### Human Attachment to Pet Versus Foster Dogs

In addition to having increased serum prolactin levels (refer to Table 10), foster dogs had carers with greater avoidant attachment toward them, compared to pet dog owners, in line with our hypothesis, refer to Table 14. We consider this to be one of the most important findings of this study because it highlights that foster dogs are being brought up by carers who are possibly reluctant to form a close attachment towards them. This is not surprising given that carers know they will eventually have to give the dogs away and so is a logical emotional defense mechanism. Avoidant attachment styles may require the dog to be more flexible from an emotional point of view and might adversely affect its development and ability to cope with subsequent rehoming experiences, which the dogs in the current study were yet to experience. Konok et al. (2015) has already demonstrated that avoidant styles of adult attachment have been associated with owning dogs with separation related disorders. While the dogs in the current study were not showing symptoms of emotional dysregulation, this could be a potentially important problem in assistance dogs given they are bred to be confident and calm. As, while they may have a natural propensity to be so, if their environment is not conducive to the development of these characteristics then consideration needs to be given as to how their environment can be enhanced and improved for their welfare and for the welfare of the humans that they are born to lead and assist.

### Limitations and Future Directions

Limitations of the present study include the uncontrolled hunger levels between subjects which may have affected motivation to perform the OCT. We tried to control for this by instructing owners/carers not to feed their dogs for 6–8 h before testing but it is possible that these instructions were not adhered to by all participating owners/carers, or that food rewards were simply not motivating enough for some dogs. Interestingly, foster dogs made more choices on the task than pet dogs and we cannot rule out the possibility that this might be due to the fact these dogs were more motivated by food than pet dogs simply because they comprised of breeds known to be more motivated by food, refer to Table 2, (Raffan et al., 2015). Hence, future studies wishing to compare assistance dogs with pet dogs should use pet dogs of the same or similar breeds. Another limitation was the set-up of the room which was not 100% symmetrical – the food was placed into the bowls behind a black screen on the dog’s left-hand side – the same side the majority of dogs with a side bias showed an overall preference for when choosing bowls. However, dogs with a significant side bias only represented a minority of the population, with 21.9% in the control trials and 25.6% in the cued trials. Collectively, in the control condition, all dogs (with and without a significant side bias) performed at chance level, which validates the experimental set-up in that they were not able to use the sense of smell to find the food, nor were they being influenced by potential subconscious “Clever Hans” effects (Pfungst, 1911) from the experimenter providing the cues. This relates to the importance of having a “blind” experimenter, a strength of the current study, so they are unable to unintentionally influence the dog’s choices according to their treatment allocation. Lastly, we chose to be consistent with the timing and dose of Oliva et al.’s (2015) previous study, however, it is currently unknown what constitutes the optimal behavioral testing time after administration of OT in dogs, and how long the behavioral effects last. However, extrapolating from the findings of a human study investigating CSF following intranasal application of 40 IU and 80 IU of the very similar peptide, vasopressin (Born et al., 2002), and a pig study investigating CSF following intranasal application of 24 IU of OT (Rault, 2016), we can reasonably assume that OT is still active in the brain 100–120 min after administration, and potentially longer. Therefore, the cognitive effects in the current study were likely to have been maintained for the entire OCT, which normally lasted between 30 and 45 min, or 75 and 90 min post intranasal administration. Lastly, our final sample size for the percentage of correct choices made was reduced due to the exclusion of dogs that made less than 10 choices for this analysis, due to the interesting finding that a number of dogs chose less than 10 times. Therefore, future studies that plan to employ a similar low level of pre-training as the current study may wish to increase the number of dogs in their sample to account for this attrition.

## Conclusion

The current study replicated previous findings that intranasal OT enhances performance on an OCT in a population of dogs that made more than 10 choices. The study furthered these findings by demonstrating that this enhanced performance is relevant to percentage correctness and not number of choices made. Furthermore, this study revealed that DAP does not have the same performance-enhancing effects. Findings that plasma OT does not increase 15 min following intranasal application of OT supports the notion that OT gains direct access to the brain, bypassing the bloodstream when administered intranasally in dogs. Neither plasma OT levels, nor serum prolactin levels were found to predict OCT performance, however, serum prolactin was found to be higher in foster dogs compared to pet dogs. Additionally, fostered dogs were more likely to perform in the OCT in terms of making a choice, but these choices were not any more likely to be correct. Fostered dogs were also more likely to be cared for by humans with an avoidant attachment toward them. These findings highlight important considerations for current assistance dog foster and training situations.

## Data Availability Statement

The datasets generated for this study are available on request to the corresponding author.

## Ethics Statement

A sample of 51 dogs and their owners or foster carers were recruited for the study. Owners/carers read through an explanatory statement before the first testing session and signed a consent form allowing their dogs to take part. The study was approved by the IRSEA Ethics Committee, approval number AFCE_201605_02.

## Author Contributions

JO, MM, AC, PP, and CF designed the study protocol. JO, MM, and TM recruited participants for the study and executed the experiments. CC carried out the biochemical analysis. ET and CL carried out the statistical analysis. JO, MM, CL, and CF analyzed and interpreted the data. JO and CF were responsible for overall administrative management of the project. JO completed the principal drafting of this manuscript. AC and PP were responsible for the funding acquisition. All co-authors were involved in re-drafting the manuscript to completion.

## Conflict of Interest

The pheromone DAP used in this study was identified and patented by PP and licensed as the commercially available product, Adaptil^®^, by CEVA Animal Health Laboratories, who provided us with both the active and sham collars used in this study. The remaining authors declare that the research was conducted in the absence of any commercial or financial relationships that could be construed as a potential conflict of interest.
